# Markov Chain Model-Based Optimal Cluster Heads Selection for Wireless Sensor Networks

**DOI:** 10.3390/s17030440

**Published:** 2017-02-23

**Authors:** Gulnaz Ahmed, Jianhua Zou, Xi Zhao, Mian Muhammad Sadiq Fareed

**Affiliations:** 1 School of Electronic and Information Engineering, Xi’an Jiaotong University, Xi’an 710049, China; gulnazahmed@stu.xjtu.edu.cn (G.A.); jhzou@sei.xjtu.edu.cn (J.Z.); 2 School of Management, Xi’an Jiaotong University, Xi’an 710049, China

**Keywords:** Markov chain-based model, optimal number, multi-hop routing, non-associated nodes, energy efficiency

## Abstract

The longer network lifetime of Wireless Sensor Networks (WSNs) is a goal which is directly related to energy consumption. This energy consumption issue becomes more challenging when the energy load is not properly distributed in the sensing area. The hierarchal clustering architecture is the best choice for these kind of issues. In this paper, we introduce a novel clustering protocol called Markov chain model-based optimal cluster heads (MOCHs) selection for WSNs. In our proposed model, we introduce a simple strategy for the optimal number of cluster heads selection to overcome the problem of uneven energy distribution in the network. The attractiveness of our model is that the BS controls the number of cluster heads while the cluster heads control the cluster members in each cluster in such a restricted manner that a uniform and even load is ensured in each cluster. We perform an extensive range of simulation using five quality measures, namely: the lifetime of the network, stable and unstable region in the lifetime of the network, throughput of the network, the number of cluster heads in the network, and the transmission time of the network to analyze the proposed model. We compare MOCHs against Sleep-awake Energy Efficient Distributed (SEED) clustering, Artificial Bee Colony (ABC), Zone Based Routing (ZBR), and Centralized Energy Efficient Clustering (CEEC) using the above-discussed quality metrics and found that the lifetime of the proposed model is almost 1095, 2630, 3599, and 2045 rounds (time steps) greater than SEED, ABC, ZBR, and CEEC, respectively. The obtained results demonstrate that the MOCHs is better than SEED, ABC, ZBR, and CEEC in terms of energy efficiency and the network throughput.

## 1. Introduction

Wireless Sensor Network (WSN) is an important technology used in different applications. The sensor nodes used in WSN are usually small battery-operated devices. These sensor nodes are installed in the field to collect the physical information. After deployment, the nodes batteries cannot be replaced. The lifetime of the WSNs ends when the batteries of these sensor nodes are empty [1]. As a result, the WSNs sensor nodes always meet a severe energy problem. The network lifetime can be increased by cutting down the usage of available resources or by energy harvesting [2,3,4,5,6]. Different schemes and strategies have been designed to reduce the energy consumption of sensor nodes. In these designed approaches, the cluster-based approaches [7,8,9,10,11,12,13,14] are the most efficient and reliable way to save the energy resources of WSNs.

The cluster-based framework is a hierarchal structure in which sensor nodes fall into a local structure called clusters [8,9,10,11,12], which consist of a Cluster Head (CH) and a few root nodes. When these root nodes are in a cluster, they are called Cluster Member Nodes $\left(C_{MNs}\right)$. The $C_{MNs}$ convey the sensed information through a CH, which manages the information of $C_{MNs}$ in its cluster by allocating transmission slots for each of them [15]. All the CHs then form the communication backbone of the network at a higher hierarchical level. In hierarchical clustering protocols [9,10,11,12], CHs perform various extra tasks compared with $C_{MNs}$, such as node association, authentication, data aggregation, data fusion, and task assignments [16]. Therefore, it is logical that the CHs would usually have much higher energy consumption as compared to the root nodes. To prevent CHs from early dying and to increase the network lifetime, the energy consumption within the network needs to be balanced by equally distributing the energy and data load among all the CHs.

The previously designed cluster-based [17,18] routing protocols randomly pick the CH node. These randomly selected CHs are not optimal in number. Consequently, this creates imbalance and uneven clusters in the network. Additionally, when the size of the cluster is greater, the pressure on CH for aggregating and receiving information also increases. The CHs which are in charge of large clusters expend more energy in comparison with the CHs of small clusters which create an unbalanced energy situation leads to end the network lifetime very earlier [19,20]. Controlling the size of the cluster is also important for balancing the load. In the proposed mechanism, we use the Markov chain model [21] to select an optimal number of CHs in the network according to the number of available sensor nodes for optimal resource utilization. In this designed framework, we also limit the number of $C_{MNs}$ in a cluster to divide the energy overhead and other CH-related responsibilities.

In current cluster-based protocols [22,23], once a sensor node gets the CH status message from all the selected CHs, in a moment, it assesses their received signal strengths. Then nodes join themselves with the CH, which has the strongest received signal strength among them [24,25]. Whenever a member node links itself to a CH which is selected on the basis of received signal strength and this CH positioned backward compared to its route of transmission towards the BS, back transmission occurs. This type of data transmission contributes to extending the general path length traveled by the locally collected data. In this paper, we present a node association strategy through which every member node compares the received signal strengths by all the CHs. If the received response by any CH is greater as compared to the other CHs, then it computes a midpoint towards the BS from both the CHs. The node then associates itself with a CH having a strong signal strength and located at a lesser distance from the BS. Otherwise, it joins the CH which is at the lesser distance from the BS.

The sensor nodes are deployed in the sensing area through a distributed algorithm [7,11]. Consequently, the distribution of nodes in the sensing field is not even. The current clustering protocols [7,8,9,10,11,12] utilize the distributed algorithm for CH selection that increase the computational overhead and also causes the resources to drain very quickly. We use Markov Bi-directional Chain Model (MBCM) [21,26] to equally divide the sensing area into clusters, and to examine the behavior of the cluster formation process. We also used this formulation [21,26] for optimal CH selection and to analyze the stochastic characteristics of the MOCHs in the sense of the mean value, the probability mass function (PMF), the standard deviation (SD), and the coefficient of variation (COV) for the optimal selection of CHs. Through this formulation [21,26], the obtained CHs are optimal in number and well-distributed all across the network, which leads to higher energy efficiency, better fairness among nodes, and prolonged the network lifetime. In our proposed clustering protocol, each sensor node consumes energy equally by revolving the CH’s responsibility between all the sensor nodes. In MOCHs, the CHs are centrally selected by the BS based on the residual energy and distance of each node from the BS. The optimal number of CHs is selected by the BS because the BS is more reliable and equipped with high-speed processors than a CH and root nodes [27]. Only a defined number of $C_{MNs}$ are joined with a CH to equally distribute the energy and data load over the network. The main contributions of our method are defined as follows:

- When the CH positioned backward compared to its direction of transmission towards the BS, back transmission occurs. This type of data transmission contributes to extending the general path length traveled by the locally collected data, which leads to a decrease the lifetime of the network. We define a simple but effective strategy for the node association to its CHs to tackle the problem of backward transmission.
- The recently designed clustering protocols [8,9,10,11,12], randomly select the CHs through a distributed algorithm which generates unequal clusters with uneven cluster size in the network; these unstable clusters create an unbalanced energy situation that leads to the end the network lifetime being much earlier. The proposed model centrally selects the optimal number of CHs by restricting the number of $C_{MNs}$ in the cluster and guarantees the longer network lifetime.
- The proposed model also deals with non-associated $C_{MNs}$ which affect the network stability and also drain the available power resources.

The remainder of the paper is organized as follows: Section 2 describes the related work and background. We discussed the methodology, the details of Markov bi-directional chain model, and motivations in Section 3. The network model, node deployment strategy and the working of the proposed clustering protocol with phases in a round are discussed in detail in Section 4. Evaluation measures and simulation results for the lifetime of the network, the network stability, the messages towards the BS, the CH selection, and the transmission time of the network are discussed in Section 5. Finally, the conclusion is drawn in Section 6.

## 2. Related Work and Background

### 2.1. Clustering

Clustering strategy reduces the redundant information by controlling the data packets transmitted and aggregated by the CHs. The clustering objective is engaged in order to improve the network performance in terms of lifetime enhancement, overhead management, fault tolerance, energy efficiency, the optimal number of CHs computation, increased connectivity, and reduced delay in data throughput. Clustering strategy can be of many types depending on different parameters. The strategies and categorization of clustering are discussed below in detail.

#### 2.1.1. Locality-Based vs. Non-Locality-Based Clustering

In locality-based clustering, usually, a sensing network is divided into cells where a single cell naturally represents a cluster. Afterward, in each cluster a set of nodes organize themselves according to their locations. Hence, in this type of clustering, every node has its location information. In a locality-based strategy, the shape of clusters can differ greatly like cross ring-shaped clusters [12], rectangular shaped clusters [22,23], and normal hexagonal clusters [28]. The cluster-based strategies which are not using location information are categorized as non-locality-based categories [7,29].

#### 2.1.2. Single-Hop vs. Multiple-Hop Clustering

In single-hop clustering, each and every node in a cluster can exchange data among themselves, and each node can communicate with its concerned CH directly [7,29]. On the other hand, in the multiple-hop strategy, a node needs to go through many hops to communicate with its concerned CH [23,30]. In this categorization, hybrid clustering is one in which the size of a cluster is changeable as follows: few clusters can be adjusted at the two-hop distance and the remaining could be adjusted to less or more hops. An example of hybrid clustering is discussed in [8], where the authors recommend larger hopping distance between far away clusters. However, there is a tradeoff between cluster organization, intra-communication and inter-communication ranges, and allocation of resources.

#### 2.1.3. Centralized vs. Distributed Clustering

There are many types of center-based clustering like [27], k-means clustering [16], k-median [23], genetic-based algorithm [25], and schemes of affinity propagation [14,31]. In center-based clustering, there exists a node that is full of resources and supervises all the clustering process. This class of clustering strategy has no scalability, which makes it unsuitable for the large scale WSNs. The other category, which is distributed clustering, is discussed in [17,19], this class of clustering is more scalable as compared to center-based clustering. However, this strategy is associated with a trade off; it involves additional signaling. Dervis et al. proposed a clustering routing algorithm which is based on the Artificial Bee Colony (ABC) algorithm [9]. The ABC’s CHs selection process is centralized. The CHs selection procedure of ABC is controlled by the BS because the hardware of the BS is more reliable as compared to the sensor node and CH. The CHs are selected on the basis of the distance of the node from the BS. The lesser the distance of the node from the BS, the greater the probability of selection as the CH. However, the nodes in [27] are selected as CH if either their energy is lesser or greater. In this way, the nodes with lesser power resources run out of battery very quickly making the network unstable.

### 2.2. Cluster Head Selection

Currently, many researchers are focusing on the problem of CH selection and CH role rotation. The CH nodes are special nodes that collect information from their member nodes and transmit it towards the BS. These nodes are in charge of a cluster and also receiving joint-request messages from different root nodes. Obviously, the selected CHs consume more energy as compared to their member nodes. That is why a lot of the previously published work [7,11,18,29] considers the residual energy of nodes as the basic parameter for the selection of CHs. In SEECH [10], the authors allow the member nodes to have the information about the average energy of the network to improve the selection criteria. These additional metrics increase overload on the network. However, the major issue here is that, as the CH task is rotated between nodes, which confirms that nodes having more energy value have the highest chance to become a CH. Therefore, this technique cannot save energy in a good way. Recently, some other parameters are also applied for the selection of CHs, like nodes location, delay, and node connectivity. In [32], a strategy is discussed for CHs selection by considering two parameters, which are node energy and delay. Centralized Energy Efficient Clustering (CEEC) [15] uses a CH selection mechanism in which CHs are selected by the BS on the basis of current location and remaining energy information from each node in the network. In [33], energy variation is taken into account by authors for selecting CHs.

### 2.3. Cluster Size

Numerous existing algorithms have presented different schemes to get an optimal cluster size in a network, where all the member nodes are distributed evenly. In [34], the authors argue that cluster sizes and intra-transmission ranges should be different in a WSN. The distance between the concerned CH and the BS is one constraint that affects the cluster size. The small size clusters are preferred at the lesser distance from the BS because the CHs nearer to the BS will transmit more data. Arranging Cluster sizes and Transmission ranges (ACT) for WSNs aims to use an equal amount of energy for each cluster by regulating the cluster size that decreases gradually from the furthest cluster to the nearest one. ACT tries to control the cluster size by regulating the radius of clusters. Another way to control the cluster size is by restricting the member nodes in the clusters. The LEACH [7,35] tries to fulfill this requirement through adjusting the number of CHs in a network. The authors define the probability value “*p*” for each node to become a CH. Therefore, the expected strength of member nodes in a cluster is defined as “1/p”.

In [36], the authors analyze the network performance through different parameters like network radius, round numbers, the length of the data packet, and consumption of the network energy. By assuming the uniform sensing nodes in the network, they compared LEACH with Optimal Energy Consumption Model (OECM) [36] and found that the CHs selection process of OECM is not stable. They considered a connection among the optimal number of CHs and assorted parameters. This method forms clusters in every round and restricts the cluster numbers by regulating the CHs in every round. However, in OECM [36] cluster formation is performed at the start of every round and information cannot be forwarded when cluster formation is in the process. So, in this way more transmission delay will occur.

To overcome the issue of longer transmission, a strategy is defined in [29]. This protocol uses multi-hop routing with a flexible transmission range of communication between clusters to overcome this issue. It selects CHs stochastically, for which rotating epoch and election probability is related to the residual energy. Nodes with higher residual energy have a higher probability of becoming CHs. However, the hot spot becomes a major issue in [29], as CHs closer to the BS will transmit more data packets. A well-balanced payload network to control data overhead through route detection and energy consumption for data transmission should be taken into account. To cope with this kind of problem, energy-efficient clustering is suggested in [13,36]. These protocols consider appropriate sizes for different clusters by looking at their hop counts towards the BS. Energy-efficient Clustering (EC) [13] successfully controls the cluster size through an adjustable probability of nodes to become a CH and to equally distribute the energy load in the network.

Our proposed protocol uses a random and an optimal CH selection scheme to manage the clusters in the sensing area. In the setup phase, the nodes estimate the location of BS and forward the sensed data at the end of each round. In previous clustering protocols [9,10,11,12,37], some distant and overpopulated CHs face heavy traffic problems and exhaust their power resources very quickly. However, the MOCHs cluster sizes are controlled by the CHs and only a restricted number of $C_{MNs}$ can join a cluster. The BS adjusts the cluster size in a manner that the more congested sensing area contains more CHs. Consequently, our designed protocol equally divides the energy load among all the CHs by dynamically rotating the role of CHs.

### 2.4. Relay Node Selection

In large WSNs, the CHs cannot forward the gathered data directly to the BS, it employs some other type of nodes to convey its data towards the BS. The other type of nodes is known as relay nodes. This type of node receives its assistant’s data, discloses this data and then conveys it in the direction of the destination (BS). An easy way to select a relay node is that CH also works as a relay node [29,37,38]. The CHs cannot be selected as relay nodes because the CHs are fulfilling two responsibilities at the same time, leading to depletion of the power resources earlier. In a randomly installed network, every node senses the data and then performs data fusion, although the normal node has limited data fusion capability. For this purpose, the location of every node is obtained via Global Positioning System (GPS) to forward the data. However, there are two issues, firstly, the bits disclosed by the normal nodes are not secure. Secondly, the inter-user carrier error propagation is a big issue. To deal with the first mention problem, secure and appropriate data aggregation is discussed in [31], which is a very challenging issue for large scale WSNs.

### 2.5. Data Routing

All the member nodes in a cluster sense data and transmit this information towards their concerned CHs. After collecting and fusing data from all the nodes the CHs transmit it towards the BS. An overlay network that only contains CHs and relay nodes is constructed in [29,39]. The traditional ad-hoc and routing protocols take into account this overlay network. A greedy data forwarding scheme is discussed in [24] which is frequently considered in previous literature for this type of routing. However, the usual problem for greedy data forwarding protocol is void zones.

## 3. Methodology

### 3.1. Problems Statement

WSNs are actually facing a range of problems, such as coverage problem, position estimation problem, security information and vulnerability problem, robustness and scalability problem, and sensors energy preservation. Concerning the energy efficiency and energy management, several routing layer protocols have been designed in [16,39]. Nevertheless, these designed protocols are not as energy efficient as required due to the following reasons:

- These protocols pick up the head nodes at random. Therefore, the selection measures of these designed protocols are poor and are able to be improved in many ways. These randomly selected head nodes are not optimal in number. Consequently, creates imbalance and uneven clusters in the network. Therefore, the CHs which are in charge of large clusters expend more amount of energy in comparison with the CHs of small clusters that creates an unbalanced energy situation leads to end the network lifetime very earlier.
- Whenever a member node link itself to a CH which is selected on the basis of received signal strength and this CH positioned backward compared to its direction of transmission towards the BS, back transmission occurs. This type of data transmission contributes to extending the general path length traveled by the locally collected data.
- These clustering protocols do not define a clear strategy for dealing with non-associated nodes which do not receive any CH joint request during the selection process. These non-associated nodes directly convey their information to BS even if they are away from CHs. To do this, a heavy amount of energy is consumed, because of which the lifetime of the network is reduced.

### 3.2. Evaluation Platform

Extensive range of simulations has been performed by MATLAB to evaluate the performance of MOCHs in comparison with Sleep-awake Energy Efficient Distributed (SEED) clustering [11], ABC [9], Zonal Based Routing (ZBR) [27] and CEEC [15] in terms of First Node Dies (FND), Half Nodes Die (HND), Last Node Dies (LND), throughput of the network, the lifetime of the network, number of CHs per round, and the transmission time of the network. The selected methods for comparison purpose are very recent in the literature of WSN and functioning of these protocols is some way associated with our proposed model. We preferred CEEC [15] and ABC [9] for comparison because the CH selection and cluster formation process of ABC are centralized and controlled by the BS. Moreover, both the protocols are trying to overcome the problem of control overhead. Despite the fact that, SEED and ZBR are selected for comparison with our proposed model for the reason that both the protocols have good cluster stability and good energy management. The average results included in these simulations have 90% confidence interval which is acquired after running the simulation 5 times. The performance comparison also reveals that the MOCHs is better than SEED [11], ABC [9], ZBR [27] and CEEC [15] in terms of energy efficiency and the network throughput.

## 4. The Proposed Clustering Protocol

In this section, first, we explain the network model of our proposed clustering protocol. We assume that there are *N* sensor nodes, which are uniformly dispersed in a square region of area 100 m × 100 m. In the literature, different techniques are used to minimize the energy consumption and also some methods are applied to find out main sources of energy drainage in the network. We believe that in the clustering routing protocol the optimal number of CHs selections also takes part in increasing the stability of the network. The size of the cluster should be controlled because sometimes the over populated clusters also lead to depleting the available resources. In our defined model, initially, all of the nodes in the network individually check their suitability for CH selection. Then the BS decides and finalizes the nominated CHs on the basis of selection criteria. The BS is working as a central entity, which reserved the rights of elimination and recommendation. We also keep a balance between the cluster size by defining a criterion in which maximum eleven and minimum six $C_{MNs}$ can jointly form a cluster. In case the maximum limit exceeds, the remainder of the nodes, joint with the other CH.

We partitioned the working of our proposed method function into rounds. Every round is then further separated into four phases such as; (1) setup phase; (2) settling phase; (3) scheduling phase and (4) data transmission phase. Whereas, the random and the optimal number of CHs selections provide a way for load balancing and the uneven energy distribution problem as well as help in keeping the stability of the network. For the third and fourth phases included in the overall energy consumption of the network, more details concerning each of the phase are explained in the next subsections.

### 4.1. Network Model of the Proposed Method

We assume a sensing network in which N sensing nodes are deployed independently and uniformly in a two-dimensional area *A(x,y)*, and the BS is located outside the field area. We are unaware about the prior knowledge of the area; in this situation, random node distribution could be a more effective strategy. In our designed WSN, we use the following random distribution algorithm to deploy the nodes in the sensing area:

(1) $$P\left(N(A)=K\right)=\frac{\left({\lambda |A|}^{K}\exp^{-\lambda |A|}\right)}{K!}$$

where *λ* is the node density and |A| is the area of the sensing field. In our designed network, nodes collect the information from the surroundings, and convey this information to the BS with the help of concerned CHs. So, in this way, there are three types of nodes in the network like field nodes, CH nodes, and the BS.

Let N is the set of field nodes, and CHs is the set of the cluster head nodes. Where, $N=\{j|j\geq 1⋀j\leq j_{\max}\}$ and $j_{max}$ is the maximum number of nodes. $CHs=\{CH|CH\geq 1⋀CH\leq CH_{\max}\}$, where $CH_{max}$ is the maximum number of CHs. Each field node is able to set up a link to the other field nodes or CH depending upon the situation. Through the above discussion, we have found this connectivity parameter:

(2) $$P_{\left(a,b\right)}=\left\{\begin{array}{c} 1\;\;\;\;\;\;\;\;\;\;\;\mathrm{If}\;a\;\mathrm{establishes}\;a\;\mathrm{connection}\;\;\mathrm{with}\;b\\ 0\;\;\;\;\;\;\;\;\;\;\;\mathrm{Otherwise} \end{array}\right.$$

The a,b∈N, and a≠b. Here, *b* is either a member node or a CH. If *b* is a CH, then *a* is a member node. The quality of links is directly related to the received signal strength and the distance.

### 4.2. Markov Chain Model for Resolving the Optimal Cluster Formation and CH Selection Problems

The nodes in the sensing field are installed through a distributed algorithm. Therefore, the distribution of nodes in the sensing field is not even. The previously designed clustering protocols [7,8,9,10,11,12] use the distributed clustering algorithm for CH selection, which increases the computational overhead on all the nodes. Another problem is that optimum numbers of CHs are also not assured through this distributed algorithm. If the selected number of CHs should not be optimal, this causes the resources to deplete very quickly. The number of CHs selected by using randomized schemes [7,8,9,10,11], is not guaranteed to be equal to the expected optimal value. To analyze the clustering properties, and to address the problems in cluster formation, we employed a bi-dimensional Markov chain model [21,26] to inspect their cluster-forming behavior. We use the Markov model to analyze the stochastic properties of MOCHs for the number of CHs in terms of the PMF, the mean, the SD, and the COV of the number of CHs. Through this formulation, the obtained CHs are optimal in number and well-distributed across the network, which leads to higher energy efficiency and better fairness among nodes, and prolonged the network lifetime.

Suppose that c(t) denotes a stochastic process to signify the selected number of CHs at a specific time instant *t*. As the process of CHs selection starts from the beginning of each round, an integer scale *t* and a discrete time t+1 instants are selected in the beginning of two successive rounds. We express that r(t) is the round at a time instant t, and x(t) is a stochastic procedure signifies the period of a scheme at a time instant t, which is x(t)=r(t)mod(1/P). We also suppose another integer 1/P, which denotes n=1/P. The state space of this model is: {0,N}⋃{(i,x):i∈[0,N], x∈[1,n−1], where,iandxareintegers}, as this process {x(t),c(t)} holds the Markov property.

To analyze the clustering properties of the proposed model, we utilize these measures: the distribution of CHs in every round, the average selected CHs in every round, the SD of the number of CHs, and the COV of the number of CHs. The target is the optimal number of CHs; the optimal number of CHs allows and guarantees the minimum energy consumption in the network. The SD calculates the variations in the target values, and the COV estimates the distribution of the average number of CHs related to the number of CHs. Let CH is a random variable indicates the total number of CHs in a round. We use the bi-directional Markov chain model stationary distribution and one-step transition probabilities [26] to estimate the PMF for the CHs as follows:

(3) $$p\left(CH=L\right)=\pi_{\left(0,N\right)}\cdot \left[P_{\left(0,N)\to (1,N-L\right)}+f_{\left(n-1,L\right)}+\sum_{x=1}^{n-2}\sum_{i=L}^{N}f_{\left(xi\right)}\cdot P_{\left(x,i)\to (x+1,i-L\right)}\right]$$

where *π* denotes staionary distribution, P is one step transition probablity matrix, and f signifies a factor matrix, $f_{xi},x\in \left[1,n-1\right],and\;i\in \left[0,N\right]$ are elements of the factor matrix. Additionally, depending upon the PMF of the number of CHs, we are able to estimate the average number of CHs (Avg[CH]), the COV (COV[CH]), and the SD (SD[CH]):

(4) $$Avg\left[CH\right]=\sum_{L=0}^{N}L\cdot p\left(CH=L\right)$$

(5) $$SD\left[CH\right]=\sqrt{\sum_{L=0}^{N}L^{2}\cdot p\left(CH=L\right)-{\left(Avg[CH]\right)}^{2}}$$

(6) $$COV\left[CH\right]=\frac{SD[CH]}{Avg[CH]}$$

Furthermore, we also consider the case if no CH is selected, then the other phases of this round will be omitted, and the next round will start. By using such enhancement, the CH selection and cluster formation process (the settling phase) becomes more effective and practical. Consequently, we exclude the cases with no CH selected in the network and we get these statistical properties given as:

(7) $$Avg_{non-zero[CH]}=E\left[CH|CH>0\right]=\frac{Avg(CH)}{1-p(CH=0)}$$

The SD and COV for the number of CHs are given as:

(8) $$SD_{non-zero[CH]}=\sqrt{\sum_{L=1}^{N}\left(\frac{p(CH=L)}{1-p(CH=0)}\right)\cdot L^{2}-{\left(Avg_{non-zero[CH]}\right)}^{2}}$$

(9) $$COV_{non-zero[CH]}=\frac{SD_{non-zero[CH]}}{Avg_{non-zero[CH]}}$$

By utilizing the Markov chain model, we perform a stage-based stochastic analysis of our model to analyze the performance of MOCHs with better granularity. The conditional PMF of the number of CHs for a stage *x* is given as:

(10) $$Pr\left(x,L\right)=\left\{\begin{array}{c} P_{\left(0,N)⟶(1,N-L\right)}\;\;\;\;\;\;\;\;x=0,L\in \left[0,N\right]\\ \sum_{i=L}^{N}f_{xi}\cdot P_{\left(x,i)⟶(x+1,i-L\right)}\;\;\;\;\;x\in \left[1,n-2\right],L\in \left[0,N\right]\\ f_{(n-1)L}\;\;\;\;\;\;\;\;\;\;x=n-1,L\in \left[0,N\right] \end{array}\right.$$

By using the Equation (10), we are able to estimate the SD, and the COV for the optimal number of CHs at each step.

### 4.3. Setup Phase

This is the first phase of our proposed model which is divided into two parts. In the first part, the initialization of the network takes place and in the second part, the neighbor discovery is completed. The details of setup phase are given below.

#### 4.3.1. Initialization

At the start of the network, the sensor nodes are randomly installed in the sensing area. During the nodes installation procedure, some points also take into consideration to avoid premature exhaustion of the network because once the nodes are employed in the field, there is no any possibility to change their batteries or their locations. The density of nodes in a specific area, the transmission distance, and the number of relaying nodes also affect the network stability.

**Figure 1:**
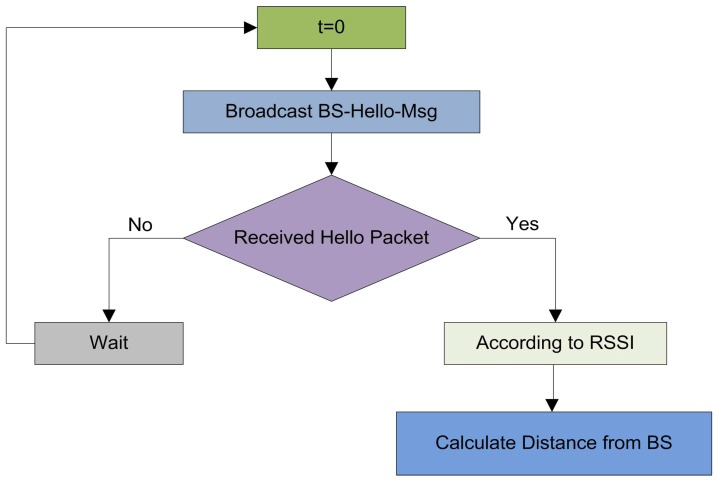
The network initialization and configuration.

In the initialization phase, the BS broadcast a BS-Hello-Msg at a certain power level. This message contains the coordinates of the BS. When this message is received by the sensor nodes, the nodes can estimate the distance of the BS according to the received signal strength indication. The illustration of the network initialization is given in Figure 1.

**Figure 2:**
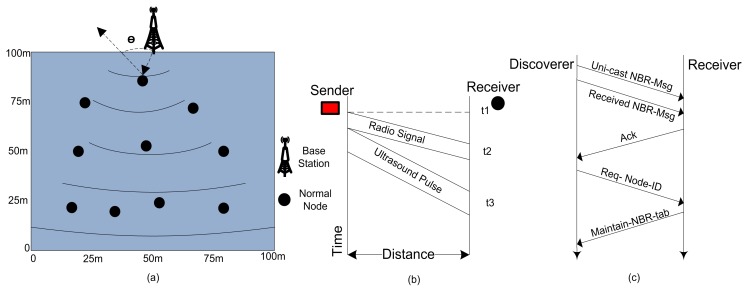
(**a**) The angle of signal arrival, (**b**) decrease in signal strength as it propagates from sender towards the receiver nodes, (**c**) neighbor discovery process.

The received signal strength indication is utilized to compute the distance of one node from another. The strength of a signal fades as long it propagates from sender nodes towards the receiver nodes as revealed in Figure 2. A radio propagation model can be used to estimate the distance between two nodes on the basis of receiving signal.

**Lemma** **1.** The initialization of the network is stabilized in finite time.

**Proof.**  The proof of this Lemma is given in Section 4 of [40] in Lemma 4. ☐

#### 4.3.2. Neighbor Discovery

**Figure 3:**
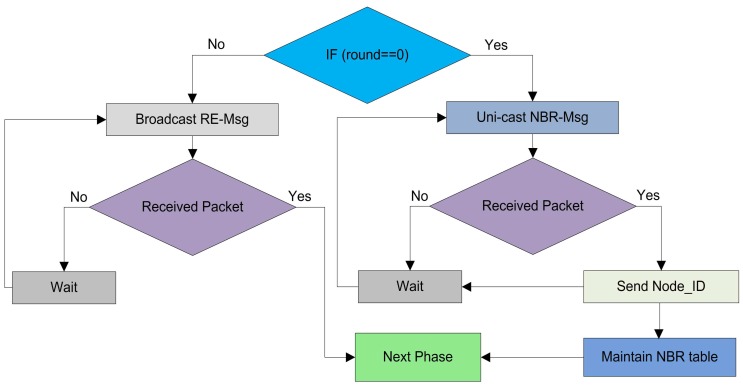
The network configuration up-gradation through neighbor discovery.

The main purpose of neighbor discovery is that in the beginning no reliable infrastructure is found among the $C_{MNs}$ for communication, and data exchange becomes crucial for WSNs. The setup phase starts with every round with the aim to upgrade the system. When all the $C_{MNs}$ in a network become familiar about some coordinates related to them, like: received signal strengths, and the node IDs, the probability of successful communications between nodes increases. We use a *NBR-Msg* exchange method to inform neighbor nodes with the node IDs, the link status, and all other coordinates of the neighbor nodes in a network as depicted in Figure 3.

The other reason for neighbor discovery is that the sensor nodes in the network decide themselves to be CHs at the beginning of every round with a certain probability. They disseminate their status as a CH Adv-CHs-Msg on intra-cluster transmission range. The nodes which are not elected as a CH receive a message from closer CHs. They send a joint request Joint-Req-Msg to concern CH. A number of sensor nodes in the network do not receive any CH status announcement from any of the CH. These sensor nodes wait for the CH status message for a predefined time slot and then these nodes become self-generating force CHs. These self-generated CHs can directly communicate with the BS either these nodes are placed close or far away from the BS. These force CHs consume additional energy during their communication to forward data. To triumph over this problem, we employ a simple strategy to handle with these non-associated nodes in our proposed clustering protocol. If a node does not receive any CH announcement message Adv-CHs-Msg it sends a request to CH through the closest neighbor. Then this non-associated node will forward its sensed data to its next neighbor node towards the BS with the help of a CH node. There is no need to send data directly to the BS. The neighbor node will forward this packet to its associated CH.

**Lemma** **2.** After the initialization, all the nodes in network discover their neighbors in finite time.

**Proof.**  All the nodes in the network spend a finite time to set the variable f(n) to themselves, here the f(n) is the initialization time. For any node j in the network, we suppose that the node j has the minimum number of neighbors i and satisfies h(i)=h(j)+g(i,j), where, the h(i) is the initialization time of node i and g(i,j) is the time taken by node i to discover j neighbor nodes. As the j has the finite number of neighbors i, then node i chooses j and sets the variable f(i) to j in a finite time. As the node i spends a finite time to set the variable f(i) of itself and at maximum waits for all the other nodes in the network to discover their neighbors in a finite time. ☐

### 4.4. Settling Phase

After the setup phase, the settling phase starts in which the whole network is divided into clusters. The details of the settling phase are given in the next subsection.

#### 4.4.1. Random CH Selection

**Figure 4:**
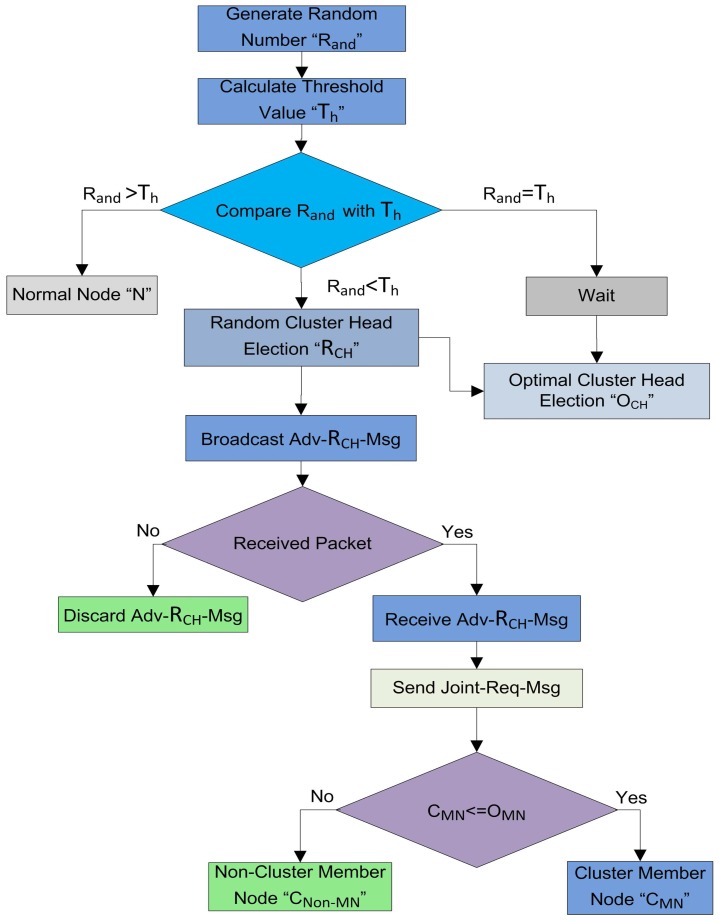
The random election, random Cluster Head (CH) selection, and random cluster formation in the network.

In this phase, every node elects itself as a local CH on the basis of certain probability. In SEED [11], every node in the network selects itself as CH on the basis of the desired percentage of CHs for the whole network. Each node chooses a random number $R_{and}$ from zero to one, then it calculates the threshold $T_{h}$. The node compares the self-generated random number $R_{and}$ with the calculated $T_{h}$. If the selected random number $R_{and}$ is less than or equal to threshold $T_{h}$, then this specific node becomes a CH for the current round. Firstly, in our proposed protocol the CHs are selected by following the random procedure. The detail of the random CH selection process of our model is shown in Figure 4.

The threshold $T_{h}$ calculating formula for MOCHs is defined as follows:

(11) $$T_{h}=\left\{\begin{array}{c} \frac{P}{1-P*(r\;mod\frac{1}{P})}\;\;\;\;\;\;\;\;\;\;\;If\;N_{i}\in \;G\\ 0\;\;\;\;\;\;\;\;\;\;\;\;\;\;\;\;\;\;\;\;\;\;\;\;\;\;\;\;\;\;\;\;\;Otherwise \end{array}\right.$$

where, *P* is the desired percentage of CHs, *r* is the current round, $N_{i}$ is a node i, and *G* is the set of nodes that have not been CHs in the previous 1/P rounds. After calculating the threshold, each node in the network generates a random number $R_{and}$ and calculates its status for the current round on the basis of the generated number. The random selection of CHs is made by each node itself on the basis of following three cases:

**Case** **1** $\left(R_{and}>T_{h}\right)$: The node cannot be designated as a CH for the current round and this node is nominated as a root node ”*N*”.
**Case** **2** $\left(R_{and}<T_{h}\right)$: In this case, the node checks whether it remained CH or not in the previous round. The node verifies its status through the list G, which contains all the names of the root nodes in the previous round. If its name is present in the list G, then it declares its status as a $R_{CH}$ for the current round.
**Case** **3** $\left(R_{and}=T_{h}\right)$: In this scenario, the node becomes a $W_{CH}$ and waits for the decision of the BS for finalizing the optimal number of $O_{CHs}$.

**Figure 5:**
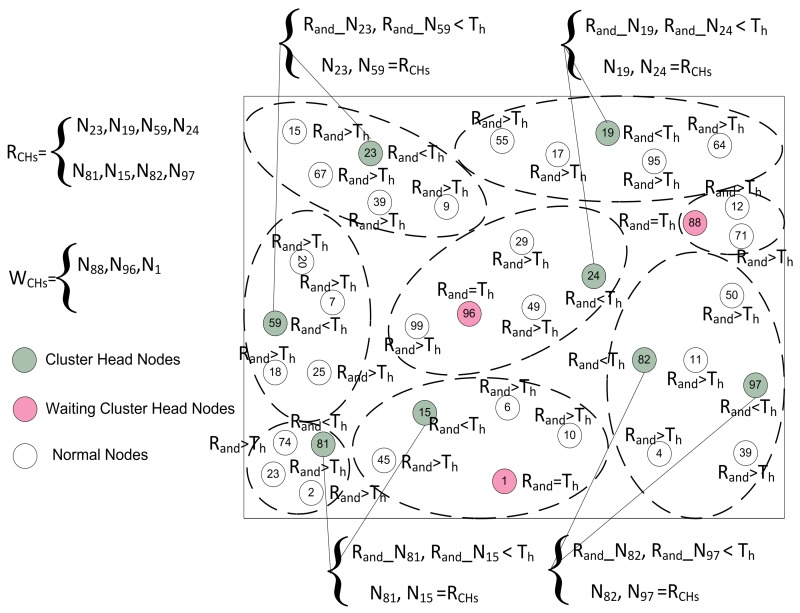
The random CH selection, and waiting for CH selection in the network on the basis of threshold.

After the random CH selection process, the nodes which are selected as $R_{CHs}$ advertise their status message as Adv-$R_{CHs}$-Msg on their intra-cluster communication range. The nodes which receive this advertisement message, send the Joint-Req-Msg to these $R_{CHs}$. When the $R_{CHs}$ receives this joint request, it checks the number of $C_{MNs}$. If the number of $C_{MNs}$ are less than the pre-defined number, then $R_{CHs}$ add this node as a $C_{MN}$ for this round. The complete random CH selection and random cluster formation procedures are defined in Figure 5.

#### 4.4.2. Optimal Number of CH Selection

**Figure 6:**
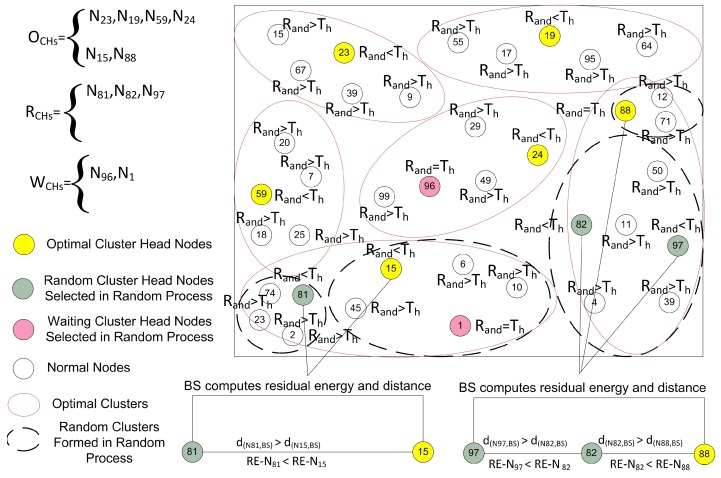
The optimal number of CH selection, and optimal number of cluster formation in the network.

Proper and careful utilization of the available resources can also help in increasing the lifetime of the network. The first step in this way is that the selection of CHs should be proper. An uneven number of CHs in every round can be a waste of resources. As defined earlier, the CH is responsible for collecting, fusing, and sending data of $C_{MNs}$. So, each step in the cluster formation consumes the power of the nodes. After the random selection of the CHs, it is essential to check whether the selected number of CHs are meeting the optimality criteria or not. In this step, the BS is involved to verify the number of CHs selected in the random selection process as demonstrated in Figure 6. The selection of CHs through a random selection process is not optimal because the CHs are selected through the distributed algorithm. So, there is a need to optimize the resources of the network. It is necessary to check if the resources are consumed in a balanced way or not. To supervise all this CH selection and cluster formation process, we engage the BS to supervise and to certify all these selection procedures. The decisions of BS are more reliable, as the BS is enriched with high-speed processors and storage capabilities as compared to root nodes [9]. The BS is not just following a single criterion, it also takes into account the distance, remaining energy, the average energy of the network, and member nodes for CH selection. Before proceeding to the next phase, the BS makes sure that the selected CHs in a random process are optimized or not. The BS calculates the optimal number of CHs through the Markov model [26] using Equation (4) according to the number of sensor nodes in the network. The BS is the central entity; it can reject an already selected CH in the random selection process as revealed in Figure 6. After that, there are three cases for finalizing the optimal number of CHs as discussed below:

**Case** **1** $\left(|R_{CHs}|>|O_{CHs}|\right)$: The BS discards some $R_{CHs}$ and all the waiting $W_{CHs}$ to achieve the optimal value.
**Case** **2** $\left(|R_{CHs}|<|O_{CHs}|\right)$: The BS selects some new $O_{CHs}$ from the waiting CHs $\left(W_{CHs}\right)$ equal to the optimal value. If the $W_{CHs}$ are not enough, then the BS can also select some new CHs equal to the optimal number from the root nodes.
**Case** **3** $\left(|R_{CHs}|=|O_{CHs}|\right)$: The BS allows the process to move to the next phase.

where, $O_{CHs}$ represent optimal CHs, $R_{CHs}$ express the randomly selected CHs, and $W_{CHs}$ express the waiting CHs. According to the first order radio energy model [11], to attain a suitable Signal to Noise Ratio (SNR) in transmitting a *l*-bit message over a distance *d*, the energy spent by the radio is given as:

(12) $$E_{TX}\left(l,d\right)=\left\{\begin{array}{c} E_{elec}l+l\varepsilon_{fs}d^{2}\;\;\;\;\;\;if\;\;d<d_{0}\\ E_{elec}l+l\varepsilon_{amp}d^{4}\;\;\;\;\;\;if\;\;d\geq d_{0} \end{array}\right.$$

**Figure 7:**
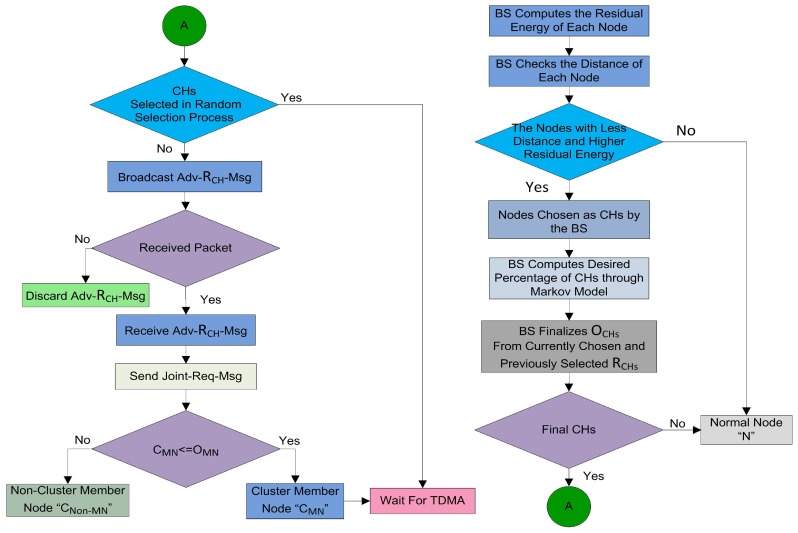
The optimal CHs selection on the basis of residual energy and distance Via BS.

Where, $E_{elec}$ is the energy consumed per bit to run the transmitter or the receiver circuit, $\varepsilon_{fs}$ and $\varepsilon_{amp}$ depend on the transmitter amplifier model we use, and *d* is the distance between the sender and the receiver. We assume that the radio channel is symmetric such that the energy required to transmit a message from node A to node B is the same as the energy required to transmit a message from node B to node A for a given SNR. The energy consumed by a CH for aggregating and sending data to the BS is computed as:

(13) $$E_{CH}=lE_{elec}\left(\frac{N}{K}-1\right)+l\varepsilon_{amp}d_{\left(CH,BS\right)}^{4}+l\varepsilon_{fs}d_{\left(N,CH\right)}^{2}$$

where, *K* is the number of clusters in a network of *N* nodes. As we discuss earlier, that the BS has unlimited resources. Therefore, these calculations and computations cannot affect the network lifetime. When the BS gets the $O_{CHs}$, then it ensures that the already selected $R_{CHs}$ are near to the optimal number or not. If the network is uniformly divided into clusters on the basis of selected $R_{CHs}$, then the BS allows the system to move on the next phase. Otherwise, it picks a few nodes with higher residual energy and lesser communication distance according to the calculated optimal number through the Markov model. In the first round, the BS selects the CHs on the basis of communication distance from the entire network. However, in the other rounds the sensor nodes, including CHs send their residual energy information with the data packets. As the rounds proceed, each node consumes energy in sensing, transmission, and reception. At the end of each round the $C_{MNs}$ and CHs calculate their remaining energy and send this information to the BS with the data packets. Now, the BS is well aware of the remaining energies of all the $C_{MNs}$ and the CHs at the end of every round. So, the BS uses this information for selecting the $O_{CHs}$ for the next round. As a result, after each round the BS updates the residual energy information of each node in the network. Finally, the $O_{CHs}$ are selected on the basis of remaining energy and communication distance. If sometimes the nodes selected through this criterion are less than an optimal number, then the CHs from the $R_{CHs}$ list and the $W_{CHs}$ list with higher residual energy are preferred to be selected as $O_{CHs}$. The optimal CH selection, and cluster formation procedures are shown in Figure 7.

#### 4.4.3. Association of Cluster Members (ACM) and Cluster Formation (CF)

In current clustering architecture, a sensor node obtains the CH status messages from the chosen CHs. It evaluates their received signal strengths. Then this node joints with a CH which has the strongest received signal strength among them. Whenever a node associates itself with a CH on the basis of receiving signal strengths, then the following major points of concern arise:

- If CH positioned backward compared to its direction of transmission towards the BS, back transmission occurs. This type of data transmission contributes to extending the general path length traveled by the locally collected data.
- The sizes of clusters become non-uniform ensuring the non-uniform overhead on the CHs.

To solve the association problems, we consider a specific scenario in which a member node *N* receives the CH status message Adv-$O_{CH}$-Msg from two CHs, e.g., $CH_{1}$ and $CH_{2}$. Then the node locates its distance $d_{\left(N,BS\right)}$ from the BS, and determines a midpoint MP. Afterward, the node compares the value of received signal from both the CHs at this midpoint. Since at this midpoint the strength of the signal from $CH_{1}$ is greater than the $CH_{2}$. As a result, the node *N* sends a joint request Joint-Req-Msg to the $CH_{1}$. The node *N* is situated at the lesser distance from $CH_{2}$ as compared to $CH_{1}$, but the node joins the $CH_{1}$ to omit the back transmission. To join with $CH_{1}$ is beneficial for the node to save the energy. To understand this, we develop the following mathematical expressions:

(14) $$d_{\left(CH_{1},BS\right)}^{2}=H^{2}+{\left(\frac{d_{\left(N,BS\right)}}{2}-X\right)}^{2}$$

where, *H* is a line joining $CH_{1}$ and a point *Q*, while *Q* is a point on the line between MP and the BS and *X* is the distance between point *Q* and midpoint MP.

(15) $$d_{\left(N,CH_{1}\right)}^{2}=H^{2}+{\left(\frac{d_{\left(N,BS\right)}}{2}+X\right)}^{2}$$

where, $d_{\left(N,BS\right)}=d_{\left(N,MP\right)}+d_{\left(MP,BS\right)}$, by adding the Equations (13) and (14), we have:

(16) $$d_{\left(CH_{1},BS\right)}^{2}+d_{\left(N,BS\right)}^{2}=2H^{2}+\frac{d_{\left(N,BS\right)}^{2}}{2}+2X^{2}$$

After substituting, $X^{2}=d_{\left(MP,CH_{1}\right)}^{2}-H^{2}$

(17) $$d_{\left(CH_{1},BS\right)}^{2}+d_{\left(N,CH_{1}\right)}^{2}=2H^{2}+\frac{d_{N,BS}^{2}}{2}+2\left(d_{\left(MP,CH_{1}\right)}^{2}-H^{2}\right)$$

(18) $$d_{\left(CH_{1},BS\right)}^{2}+d_{\left(N,CH_{1}\right)}^{2}=2H^{2}+\frac{d_{\left(N,BS\right)}^{2}}{2}+2\left(d_{\left(MP,CH_{1}\right)}^{2}\right)-2H^{2}$$

(19) $$d_{\left(CH_{1},BS\right)}^{2}+d_{\left(N,CH_{1}\right)}^{2}=\frac{d_{\left(N,BS\right)}^{2}}{2}+2\left(d_{\left(MP,CH_{1}\right)}^{2}\right)$$

We can note from Equation (18) that the distance of the node from BS $d_{\left(N,BS\right)}$ is constant, and $d_{\left(CH_{1},BS\right)}^{2}+d_{\left(N,CH_{1}\right)}^{2}$ is directly related to $d_{\left(MP,CH_{1}\right)}$. If we minimize this $d_{\left(CH_{1},BS\right)}^{2}+d_{\left(N,CH_{1}\right)}^{2}$, then, we can attain our objective which is equivalent to $Min(d_{\left(MP,CH_{1}\right)})$. Consequently, if a member node selects the CH closer to the midpoint towards the BS, than the squared distance of their communication is smaller, which means that the energy utilization is minimized for that node, leading to increasing the network lifetime. The energy consumption of a node in a cluster formation process is:

(20) $$E_{N-CF}=2lE_{elec}+l\varepsilon_{fs}d_{\left(N,CH\right)}^{2}$$

So, the total energy consumed for dividing the whole network into clusters is:

(21) $$E_{CF}=K\left[E_{CH}+\left(\frac{N}{K}-1\right)E_{N-CF}\right]$$

The WSN nodes have inbuilt resource limitations, so, the clustering procedure is commonly adopted for WSN applications to accomplish the energy efficiency. Clustering is an efficient method to organize the WSN nodes into hierarchal groups. This hierarchal structure is adopted at different layers like the Network layer or the Data Link layer according to the system requirement. Clustering improves the system performance by reducing the local network traffic, the long-distance communication, and the routing information of root nodes. The proposed model also adopts clustering due to the following reasons:

- Clustering facilitates in reducing the cost of topology maintenance as a reaction to dynamic topology changes.
- In a clustered structure, the topology reconfiguration is only performed on the CH level and it does not affect root nodes.
- Clustering also minimizes the overhead generated due to dynamic topology adaptation.
- Clustering allows resource utilization optimization which is successfully used to save time and energy.

**Lemma** **3.** The time and data packet exchange complexity of MOCHs for cluster formation in a round is O(1). While the time and the message exchange complexity of the proposed scheme for dividing the whole network into random clusters during a round with N number of nodes is O(N).

**Proof.**  After the selection of optimal number of CHs, the election process is completed. Each of the sensor nodes either sends or receives a message. The nodes which are selected as CHs for this round advertise their status as CHs, while the $C_{MNs}$ send a Joint-Req-Msg to their associated CHs merely. Therefore, the data packet exchange complexity of a node during a round is O(1). Thus, the information exchange complexity of the proposed model for developing clusters in a round is O(N). In the cluster formation procedure, each sensor node in the sensing field needs to process N−1 nodes despite the fact that in severe cases to become a $C_{MN}$. This entire process is completed in a predefined time slot. The time complexity of a round for selecting a CH is the O(1). Consequently, the time complexity of cluster formation in a round of the proposed scheme is O(N). ☐

**Lemma** **4.** The time complexity of MOCHs for selecting a CH in a round is O(1). While the time complexity of the proposed scheme for the whole network with N number of nodes is O(N).

**Proof.**  In a random CH selection process of MOCHs, all the nodes in the network generate a random number $R_{and}$ between 0 and 1. Then these nodes compare this generated number $R_{and}$ with the threshold value $T_{h}$, pre-computed by employing the Equation (10). If the randomly generated number $R_{and}$ of a node is less than the threshold value computed by Equation (10), in this case that node elects itself as a CH for this current round. This entire course of action for random CH selection is completed during a predefined time slot. Consequently, the time complexity of a single node for selecting it as a CH is O(1). Accordingly, the time complexity of selecting CHs in a round for the proposed model is O(N). ☐

#### 4.4.4. Dealing with Non-Associated Cluster Members (NACM)

**Figure 8:**
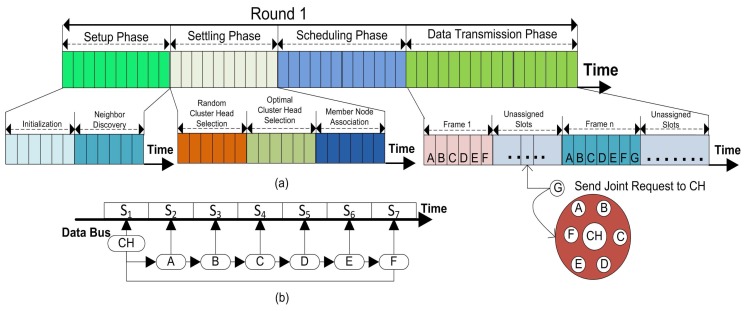
(**a**) The complete description of phases in a round of model-based optimal cluster heads (MOCHs) and the association process of non-cluster member for data forwarding; (**b**) CH data collection during a round in slots.

Some sensor nodes in the network are not receiving a CH advertisement message from any CH. These nodes wait for a specific time slot for the CH advertisement message and after that predefined time slot, these nodes start sending their data directly to the BS. In the literature of WSN [7,8,9,10,11], these self-generated CHs are known as forceclusterheads. These self-generated force CHs are a waste of system resources, because all the time in a round these force CHs repeatedly send their information to the BS which affects the network stability. We also define a strategy to deal with these force CHs and we named them Non-Associated Cluster Members (NACM). In MOCHs, if a node does not receive any CH advertisement message, it will send a data forwarding request to the CH of its closest neighbor node. The requested CH will assign a data slot for this NACM node at the request. Then the neighbor node will forward the data of this NACM node during the assigned slot as shown Figure 8. The energy consumption of a NACM node to forward a data packet of *l* bits is:

(22) $$E_{NACM}=2lE_{elec}+l\varepsilon_{fs}d_{\left(N,NBR\right)}^{2}$$

Here, $d_{\left(N,NBR\right)}^{2}$ is the distance between a field node and its neighbor (NBR). The energy consumption of a neighbor node to receive a data packet of *l* bits is:

(23) $$E_{NBR}=lE_{elec}$$

### 4.5. Scheduling Phase (SP)

Once all the sensor nodes are structured into clusters, each CH creates a schedule for the $C_{MNs}$ in its cluster. This allows the radio components of each $C_{MNs}$ to be turned off during all the time slots except its transmit time, thus this action minimizes the energy dissipated by the individual sensors.

Thus, overall energy consumption in SP is computed as in [11]:

(24) $$E_{SP}=K\left[E_{CH-SP}+\left(\frac{N}{K}-1\right)E_{N-SP}\right]$$

There are two modes of communication like Ready-to-receive and Time Division Multiple Access (TDMA). In the Ready-to-receive mode, the nodes remain active all the time to send its data. Thus consumes a lot of energy resources. While in the second mode, the nodes, after receiving their time slots, turn into sleeping mode and set the right time to awake for communication. In the sleeping mode, the sensor nodes save a lot of energy. We adopted the TDMA mode for our proposed model because it saves the energy resources and also it is designed for communication over shortest path on established links in awake up mode.

**Lemma** **5.** In the MOCHs, TDMA slot allocation time and message exchange complexity are O(1) for each node and for the entire network with $\left(N-O_{CHs}\right)$ nodes is $O(N-O_{CHs})$. Where N is number of member nodes and $O_{CHs}$ is the set of optimal number of CHs.

**Proof.**  After cluster formation process, each CH in the network sends a TDMA slot to each member node. So, the message exchange complexity of one node is O(1). Therefore, the message exchange complexity for TDMA slot allocation in MOCHs algorithm is $O(N-O_{CHs})$. After receiving this TDMA schedule each $C_{MN}$ sends an acknowledgment message to the CH. This whole procedure is done during a predefined time period. The time complexity of one node is O(1). Thus, the time complexity for TDMA slot allocation in MOCHs algorithm is $O(N-O_{CHs})$. ☐

### 4.6. Data Transmission (DT) phase

The data transmission phase starts after the time slots are allocated to every associated $C_{MNs}$ in the network. This phase is the most important phase in the lifetime of the network because nodes in this phase send their sensed data to their relevant CH which aggregates and forwards this correlated data to the BS. The total energy consumed by all the nodes for transmitting and receiving a full-length data packet containing *F* frames is calculated in a similar way as in [11]:

(25) $$E_{DT}=K\left[E_{CH-DT}+\left(\frac{N}{K}-1\right)E_{N-DT}\right]\times F$$

After computing the energy consumed in each phase of a round, finally, we calculate the energy consumed by all the nodes during a complete round as:

(26) $$E_{round}=E_{CF}+E_{SP}+E_{DT}\times F$$

In recent cluster-based protocols [9,10,12,27], the CHs convey their data to the BS through relay nodes. Most of the times the CHs towards the BS are chosen as relay nodes. In this case, the CHs sensing their area and also working as the head nodes. The CHs are ordinary nodes with no extra resources and when these ordinary nodes are working as CHs their energy consumption increase. However, when these nodes are selected as relay nodes to forward the data of backward CH nodes, these nodes exhaust their power resources very quickly. The CH nodes, which are closer to the BS are most of the time run out of batteries making the network unstable much earlier. In MOCHs, the CHs collect all the information from their $C_{MNs}$ and then they directly convey collected information to the BS.

**Lemma** **6.** All the sensed data packets in the network sensed by the $C_{MNs}$ arrive at the CHs.

**Proof.**  The root nodes, which receives a CH status message Adv-$O_{CHs}$-Msg, send their Joint-Req-Msg to the concern CHs. The CHs then accept their request and these root nodes become the $C_{MNs}$. After cluster formation, the CH sends a TDMA slot to each node individually in which $C_{MNs}$ send their packets to the CHs. So, each member node in the network sends one data packet to the CH. Hence, all the nodes in the network send all the sensed data packets to the CHs. ☐

## 5. Performance Evaluation

We execute a series of simulations to analyze and evaluate the performance of MOCHs. We presume that all the sensor nodes deployed in the sensing field have the same size of data, the data of block size are 4000 bits. We take network size of 100 m × 100 m in which 100 nodes are randomly distributed. The BS is placed outside the sensing area in any arbitrary position. We take different scenarios in which nodes with different intensity are deployed in the network. We also take different initial energies of the sensor nodes to check the strength and bounds of our designed framework. We used five different evaluation measures which are very famous in WSN literature and employed to check the performance of clustering protocols. The quality measures that we engaged in the evaluation are: the lifetime of the network, stable and unstable region in the lifetime of the network, throughput of the network, the number of CHs in the network, and the transmission time of the network. Simulation parameters used for our experiments are given in Table 1 and the performance measures used for simulations are described in the next subsection.

### 5.1. Performance Measures

These terms are used in SEED [11], we also use these terms to evaluate and analyze the performance of our proposed clustering protocol.

- Network lifetime has been proved a very important paradigm in WSNs literature. It is the time span of both stable and unstable regions of the network. In this time duration, all the nodes in the network deplete their energy resources.
- From the beginning of the network operation up to the first node depletes its battery is the stable region of the network. This is the time span in which a network generates maximum throughput, as the generated data has direct proportion with the alive number of nodes.
- The timeline starts when the first node runs out of battery up to all the sensor nodes deplete their batteries is called the unstable region in the lifetime of the network. Generally, in this time duration throughput of a network gradually decreases.
- The evaluation metric for the number of CHs also plays very important role in WSNs. This selection measures means that the selection of CHs should be optimal, as, proper and careful utilization of the available resources can help in increasing the lifetime of the network.
- Transmission delay is a time period for which a complete data packet is successfully received at the BS.
- Data throughput is also an important metric for WSN. The successful delivery of the data packets at the BS is called the throughput of the network. There is always a tradeoff between the network lifetime and the throughput of the network. The greater the lifetime of the network, the greater the throughput and vice versa.

The details of these performance metrics are described in detail in the next subsections.

### 5.2. Analysis of Our Proposed Model

To deeply investigate the clustering characteristics and cluster formation behavior of our model, we derive formulations using the stochastic properties of our model for the number of CHs in terms of the SD, and the COV in the selection of CHs. This formulation is very helpful in designing and selection of optimal CHs for our proposed model.

#### 5.2.1. The Standard Deviation, and the Coefficient of Variation of the Number of CHs

**Figure 9:**
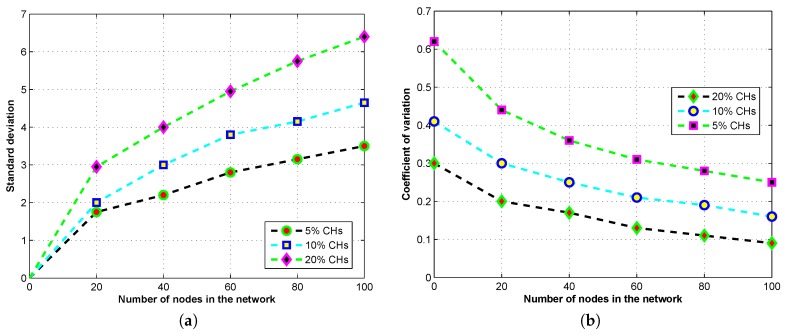
The number of clusters and CHs analysis through standard deviation, and the coefficient of variation. (**a**) The standard deviation of CHs in the network; (**b**) The coefficient of variation of CHs in the network.

The current clustering schemes are using distributed methods for CHs selection which do not assure the optimal value of CHs according to our analysis. We also found that the optimum percentage of CHs is always less than 20% which does not affect the network lifetime. In worst case scenarios, when no CH is selected or the CHs are less than the optimal value the clustering structure will collapse or the network will drain its resources earlier than expected. The unevenness of CHs in the clustering structure badly affects the energy efficiency and the network lifetime. Figure 9a,b demonstrates the COV and the SD in the selection of CHs; the results are extracted from the proposed model in different scenarios. We can see that the larger the sensing field the greater the number of CHs, and the wider the distribution for the number of CHs.

#### 5.2.2. Effect of Random and Optimal CH Selection on Our Proposed Model

**Figure 10:**
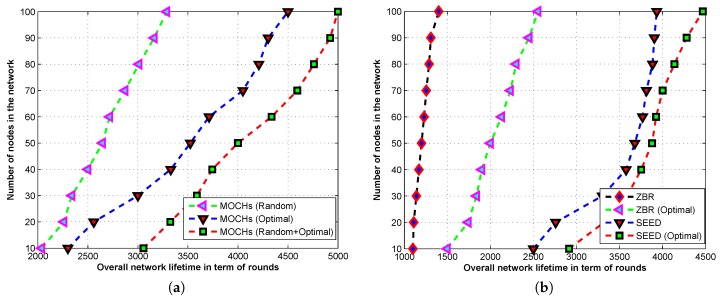
Analysis of random and optimal CHs selection on the lifetime of ZBR, SEED, and the proposed algorithm. (**a**) The effect of random and optimal CHs selection on the lifetime of MOCHs; (**b**) The effect of optimal CHs selection on the lifetime of ZBR, and SEED.

To check the suitability of optimal and random CHs of the proposed model, we perform simulations in different scenarios in which the proposed model selects the random, optimal, and random and optimal CHs. When the proposed model selects the CHs randomly, it uses the distributed algorithm to select the CHs. In this case, sometimes the numbers of CHs are near optimal. However, most of the time, the selected CHs are not optimal and consume the network resources, which rapidly leads to ending the network lifetime earlier. However, the optimal number of CHs selection is only possible with the help of the BS. Due to the BS selection, the nodes do not use their energies for computation and communication. So, the network saves a lot of energy through optimal CH selection which leads to a greater lifetime. However, the BS’s CHs selection is dependent on residual energies and distance from itself. The nodes with higher energies and at the lesser distance are selected while the member nodes suffer backtracking or link breakage due to the unsuitability of received messages from CH nodes. Conversely, this problem is solved when the proposed model simultaneously uses the optimal and random CHs selection as depicted in Figure 10a. We also check the effect of optimizing the CHs selection process on ZBR and SEED. Both these protocols use distributed algorithms for CHs selection and suffer a lot due to uneven energy consumption. After optimal CHs selection, we can see the increment in the lifetime of both the models in Figure 10b.

### 5.3. Comparison with State-of-the-Art Methods

To evaluate the performance of our proposed mechanism we selected few state-of-the-art protocols like SEED [11], ABC [9], ZBR [27] and CEEC [15] for comparison purposes. The selected methods are very recent in the literature of WSN and working of these protocols is somehow related to our proposed model. We preferred CEEC [15] and ABC [9] for comparison because the CH selection and cluster formation process of ABC are centralized and controlled by the BS. Moreover, both the protocols are trying to overcome the problem of control overhead. The SEED and ZBR are selected for comparison with our proposed model because both the protocols have good cluster stability and good energy management. For a fair comparison, all the conditions such as the initial network energies, the simulation environment, the node distribution, and the node densities in the network are taken same for all the methods. The average results included in these simulations have 90% confidence interval which is acquired after running the simulation 5 times.

#### 5.3.1. The Lifetime of the Network

**Figure 11:**
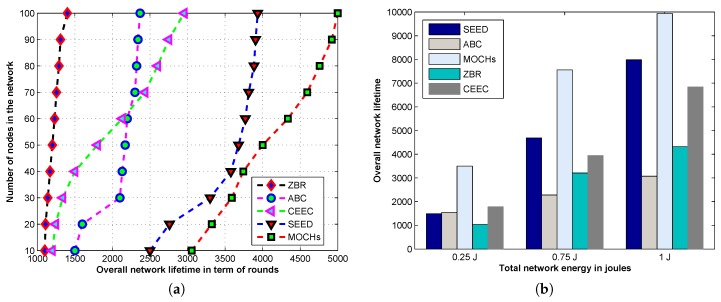
The lifetime comparison of MOCHs with different protocols in terms of rounds with different initial energies of the deployed sensor nodes. (**a**) The analysis of the network lifetime with the number of alive nodes; (**b**) The evaluation of the network lifetime with different energies.

Figure 11a illustrates the lifetime comparison of the proposed model against the state-of-the-art clustering protocols like SEED, ABC, CEEC, and ZBR. Here, we discuss the lifetime of the network, which is defined as the time interval in which all the sensor nodes in the network drain their batteries. All the sensor nodes in the network of MOCHs, SEED, ABC, ZBR, and CEEC run out of batteries at about 4999, 3934, 2369, 1400, and 2954 rounds, respectively. The lifetime of the proposed model is approximately 1095, 2630, 3599, and 2045 greater than SEED, ABC, ZBR, and CEEC, respectively. The lifetime of the proposed model is approximately 22%, 53%, 72%, and 41% rounds greater than SEED, ABC, ZBR, and CEEC, respectively. The lifetime of MOCHs is 22% greater than SEED because the CH selection criterion is marginally better in SEED, which restricts the number of the cluster to a certain limit due to that no extra CHs are selected. In the clustering protocols, the good selection criterion for the CHs selection saves a lot of energy in the network. The cluster formation mechanism is distributed in three different zones, no extra time and energy are consumed for repeated CH selection and cluster formation. In MOCHs, the optimal CH selection is done by the BS which contains unlimited resources and helps to save the energy of the network. The cluster formation involves a lot of processing and consumes the energy of the network. In SEED this selection is completed by CH and consumes almost 20% of the network resources, which decreases the lifetime of SEED. The lifetime of ZBR is 72% lesser than the proposed model because in ZBR the multi-hop communication is used to forward the data from the CH to the BS. The BS is placed outside the sensing field in any arbitrary place. The CHs closer to the BS relay the data of almost 3 back clusters. Consequently, the CHs closer to the BS run out of battery very quickly compared to the distant CHs which make the network unstable. Figure 11b depicts the network lifetime of MOCHs, SEED, ABC, CEEC, and ZBR with different network energies like 0.25 J, 0.75 J, and 1 J. We can see that the proposed models’ performance also remains very good at different network energies. We also compare the lifetime of the network with varying the node distribution. The Table 2 and Table 3 demonstrate the comparison of the lifetime of the network with N=200 and N=300. From the tables, we can see that the proposed model outperforms as compared to SEED, ABC, CEEC, and ZBR in all the distribution scenarios.

#### 5.3.2. The Stable and Unstable Regions in the Lifetime of the Network

**Figure 12:**
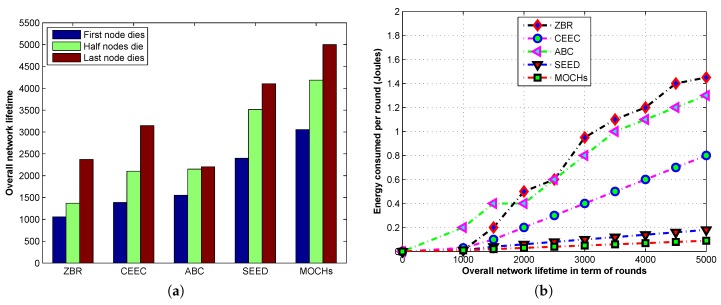
The stable and unstable regions comparison of MOCHs with SEED, CEEC, ABC, and ZBR in the lifetime of the network. (**a**) The network stability comparison; (**b**) The analysis of energy consumption per round.

The stability period is defined as, the time interval that begins when the first node depletes its battery. The Figure 12a depicts the comparison of stable and unstable regions of the proposed model with SEED, CEEC, ABC, and ZBR. The first nodes of the MOCHs, SEED, CEEC, ABC, and ZBR drain their batteries at about 3055, 2500, 1200, 1500, and 1100 rounds, respectively. The stability period of the proposed model is approximate 19%, 61%, 51%, and 64% greater than SEED, CEEC, ABC, and ZBR, respectively. The stability period is the time duration in which all the sensor nodes are alive and the performance of the network is maximum. The proposed model has very long stable period, because of the good energy management and good energy distribution among all the dynamic clusters. After that, the unstable region starts with the death of the first node. In an unstable region, the nodes run out of batteries and the performance of the network decreases gradually. The unstable period of the proposed model in comparison with SEED, CEEC, ABC, and ZBR is about 1945, 1434, 1754, 869, and 200 rounds greater, respectively. In ZBR due to multi-hop communication, the CHs closer to the BS run out of battery soon compared to the distant CHs. When the CHs closer to the BS deplete their batteries the network un-stabilizes for some time and collapses after 200 rounds. However, in SEED, ABC, and CEEC after the first node run out of battery and the network becomes unstable for the lifetime of the network due to uneven energy distribution. Figure 12b illustrates the network energy consumption of MOCHs, SEED, CEEC, ABC, and ZBR. The energy consumption of the proposed model is optimized and remains smooth in the whole network lifetime. The energy consumption is very low because MOCHs use the available power resources in very optimized and balanced way which increases its lifetime. The energy consumption of SEED is also balanced due to its even energy distribution in the network. The Table 4 and Table 5 reveal the comparison of the stable and unstable regions of the network with N=200 and N=300. From the tables, we can see that the proposed model’s performance remains remarkable with different node densities as compared to SEED, CEEC, ABC, and ZBR.

#### 5.3.3. The Throughput of the Network

**Figure 13:**
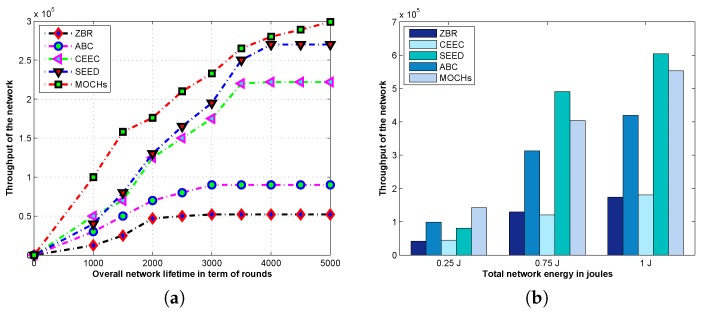
The total network throughput comparison of the proposed model against SEED, ABC, CEEC, and ZBR in the lifetime of the network on the basis of initial energies of the node. (**a**) The network throughput analysis; (**b**) The evaluation of network throughput with different initial energies.

The successful delivery of the data packets at the BS is called the throughput of the network. There is always a tradeoff between the network lifetime and the throughput of the network. The greater the lifetime of the network, the greater is the throughput and vice versa. The Figure 13a describes the comparison of network throughput of the proposed model against SEED, ABC, CEEC, and ZBR. The throughput of MOCHs, SEED, ABC, CEEC, and ZBR is 2,990,000, 2,700,000, 900,000, 2,220,000, and 520,000 data packets, respectively. The throughput of the proposed model is 290,000, 2,090,000, 770,000, and 2,470,000 data packets greater than SEED, ABC, CEEC, and ZBR, respectively. The proposed model has 10%, 70%, 26%, and 82% greater throughput than SEED, ABC, CEEC, and ZBR, respectively. As we discussed earlier, the network with the greater lifetime has the greater throughput. The proposed model has 1095 rounds greater lifetime than SEED; therefore, the proposed model has greater output than SEED. The throughput of the CEEC, and ABC is persuasive due to the longer lifetime. Both these schemes are using the centralized CHs selection methods and saving a lot of network energy, which leads to a longer network lifetime with a greater throughput. While in ZBR the network depletes its resources earlier than expected due to the CHs closer to the BS. The nodes closer to the BS selected as CHs and also working as relay nodes. When these nodes run out of batteries the lagged behind nodes cannot convey their sensed information to the BS and the network collapse resulting a much lower throughput. Figure 13b portrays the network throughput of SEED, ABC, CEEC, and ZBR with different network energies like 0.25 J, 0.75 J, and 1 J. The proposed model performance remains very persuasive with different network energies such as 0.25 J, 0.75 J, and 1 J. We also compare the throughput of the network with varying the node densities. The Table 6 and Table 7 reveal the comparison of the throughput of the network with N=200 and N=300. From the tables, we can notice that the proposed model outperforms as compared to all of the above discussed methods.

#### 5.3.4. The Number of CHs in the Network

**Figure 14:**
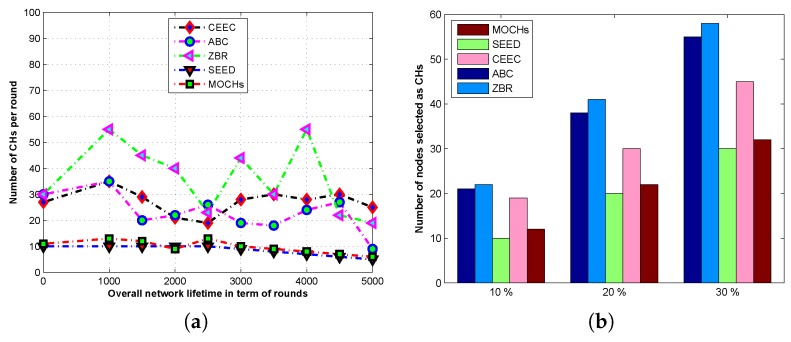
The comparison of total number of CHs selected per round in the lifetime of the network of MOCHs with different protocols. (**a**) The analysis of number of CHs per in the network; (**b**) The effect of different of number of CHs on the network.

The nodes in the sensing field are installed through the distributed algorithm. Therefore, the distributions of nodes in the sensing field are not even. The previously designed clustering protocols use the distributed clustering algorithm for CH selection, which increases the computational overhead on all the nodes. Another problem is that the optimum numbers of CHs are also not guaranteed through this distributed algorithm. If the selected number of CHs is not optimal, this causes the resources to deplete very quickly. In this proposed model, we introduce a new mechanism which restricts the algorithm to select the optimal number of CHs in each round. Figure 14a depicts the comparison of the number of CHs selected per round in SEED, ABC, CEEC, and ZBR. The proposed model always chooses the optimal number of CHs in comparison with selected state-of-the-art clustering protocols. While the other clustering protocol selects the CHs through distributed algorithm, so, their selection criterion is not very good. Consequently, the number of CHs in ZBR and ABC vary from 10%–50% CHs per round during the lifetime of the network. The performance of our algorithm remains very consistent when we increase the node densities from N=100 to N=300, it always meets the optimality criterion for CHs selection as depicted in Figure 14b.

#### 5.3.5. The Transmission Time of the Network

**Figure 15:**
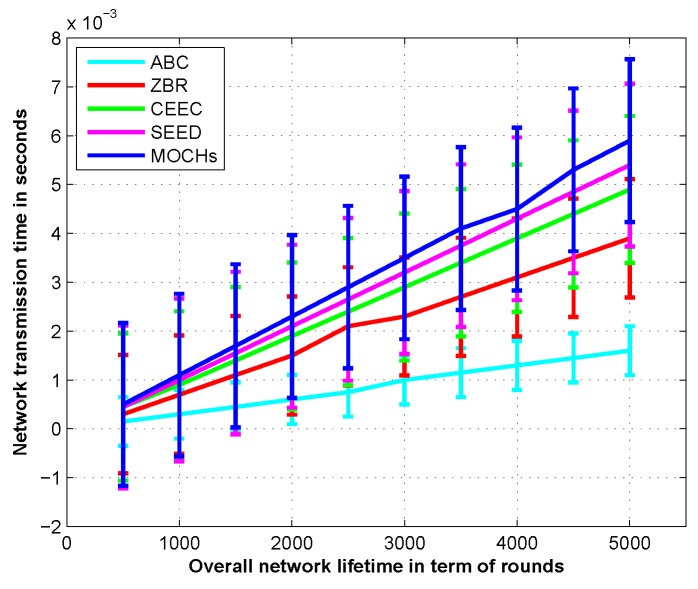
The transmission time in the life cycle of the network with different density of the nodes in the sensing field.

The time span from the establishment of the network up to the last data bit of the data has left the transmitting node. Figure 15 depicts the comparison of the transmission time of the proposed model with SEED, ABC, CEEC, and ZBR. Better packet sending rate of the proposed protocol causes an increase in the average transmission delay per packet as compared to ABC, CEEC, and ZBR.

## 6. Conclusions

In this paper, we have designed a centralized protocol in which the BS has complete authority to supervise and restrict the cluster formation criteria to meet optimality. Our clustering protocol also manages the non-associated cluster members very well, which can affect the network stability region and exhaust the available resources. The most important purpose of the proposed clustering protocol is to come up with a new modified protocol design to tackle the problem of backward transmission. MOCHs can guarantee the longer network lifetime with minimum energy utilization at the same time taking other performance criteria into consideration. We analyze our model using five different measures and perform a wide-range of simulations to validate our model. The simulations are also performed with different node densities to check the performance of our model. The extracted results demonstrate that MOCHs has longer network lifetime and more stable clustering as compared to the state-of-the-arts methods. In future work, MOCHs can be further enhanced by taking into consideration the energy harvesting scheme to increase the network lifetime.

## Figures and Tables

**Table 1:** Parameters and their values used in simulation environment.

Variable	Value
Size of network	100 m × 100 m
N	100
$E_{elec}$	50 nJ/bit
$\varepsilon_{amp}$	100 pJ/bit/m${}^{2}$
$\varepsilon_{fs}$	10 pJ/bit/m${}^{2}$
$E_{o}$	0.25 J, 0.5 J, 0.75 J
$E_{DA}$	5 nJ/bit/message
$P_{opt}$	0.1

**Table 2:** Network lifetime over simulations of five Wireless Sensor Networks (WSNs) (with 200 nodes).

WSN#	ABC [9]			CEEC [15]			SEED [11]			MOCHs		
	FND	HND	LND	FND	HND	LND	FND	HND	LND	FND	HND	LND
1	1110	1510	2156	1048	1934	3047	2372	3631	3724	2957	3960	4927
2	1121	1571	2202	1090	1950	2869	2199	3421	3702	2973	3884	4990
3	1094	1522	1977	1088	1920	2966	2299	3538	3697	2859	3876	4962
4	1067	1540	2209	1135	1926	3005	2295	3394	3637	3004	3899	4952
5	1130	1512	2173	1099	1987	2881	2229	3492	3705	2911	3968	4971
**Average**	1104.4	1531	2143.4	1092	1943.4	2953.6	2278.8	3495.2	3693	2940.8	3917.4	4960.4

**Table 3:** Network lifetime over simulations of five WSNs (with 300 nodes).

WSN#	ABC [9]			CEEC [15]			SEED [11]			MOCHs		
	FND	HND	LND	FND	HND	LND	FND	HND	LND	FND	HND	LND
1	1049	1516	2029	1039	1898	2960	2346	3524	3756	2743	3754	4724
2	1093	1533	2109	1074	1925	2873	2370	3545	3720	2764	3671	4809
3	1109	1436	2080	1029	1869	2866	2340	3485	3694	2798	3711	4754
4	1155	1428	1996	1055	1864	2940	2256	3408	3729	2801	3746	4766
5	1098	1423	1963	1012	1857	2870	2255	3408	3662	2721	3696	4707
**Average**	1100.8	1467.2	2035.4	1041.8	1882.6	2901.8	2313.4	3474	3712.2	2765.4	3715.6	4752

**Table 4:** Round history of dead nodes over simulations of five WSNs (with 200 nodes).

% Dead Nodes	ABC [9]	CEEC [15]	SEED [11]	MOCHs
10	1283	1357	2675	2955
20	1347	1541	2952	3205
30	1387	1700	3158	3527
40	1472	1825	3399	3611
50	1510	1934	3631	3853
60	1576	2022	3720	4147
70	1652	2133	3722	4471
80	1701	2264	3722	4598
90	1866	2372	3723	4761
100	2156	3047	3724	4909

**Table 5:** Round history of dead nodes over simulations of five WSNs (with 300 nodes).

% Dead Nodes	ABC [9]	CEEC [15]	SEED [11]	MOCHs
10	1290	1330	2601	2906
20	1372	1475	2784	3143
30	1431	1610	2997	3468
40	1467	1727	3226	3551
50	1516	1898	3485	3782
60	1568	2034	3677	4103
70	1656	2176	3692	4387
80	1750	2296	3693	4511
90	1827	2437	3694	4696
100	2029	2960	3694	4829

**Table 6:** Total number of data packets received at the BS over simulations of five WSNs (with 200 nodes).

WSN#	ABC [9]			CEEC [15]			SEED [11]			MOCHs		
	FND	HND	LND	FND	HND	LND	FND	HND	LND	FND	HND	LND
1	23,330	30,204	37,727	40,771	73,978	89,037	431,970	606,156	613,866	197,382	250,314	611,514
2	24,999	33,014	39,575	40,651	72,455	89,405	399,194	565,777	587,633	191,021	250,912	619,415
3	21,315	28,074	33,601	39,979	70,099	87,547	418,648	590,185	602,940	181,715	251,178	621,472
4	21,908	29,651	36,691	41,530	70,391	87,271	418,284	564,967	583,522	198,771	252,224	615,609
5	22,830	29,135	36,040	40,723	74,860	90,035	403,357	577,858	593,654	193,534	248,468	613,107
**Average**	22876.4	30,015.6	36,726.8	40,730.8	72,356.6	88,659	414,290.6	580,988.6	59,6323	192,484.6	250,619.2	616,223.4

**Table 7:** Total number of data packets received at the BS over simulations of five WSNs (with 300 nodes).

WSN#	ABC [9]			CEEC [15]			SEED [11]			MOCHs		
	FND	HND	LND	FND	HND	LND	FND	HND	LND	FND	HND	LND
1	31,494	42,207	49,193	48,364	80,649	101,703	650,466	898,763	926,996	220,614	360,155	888,213
2	33,945	44,467	52,345	48,945	79,702	99,044	658,340	899,517	919,863	227,300	363,950	897,446
3	32,281	39,644	48,860	47,055	77,852	97,350	647,950	882,923	907,599	228,851	369,314	903,309
4	31,257	40,024	48,750	50,787	81,261	102,020	623,863	866,588	903,315	237,280	364,807	894,681
5	32,595	39,911	48,487	46,953	77,664	98,608	616,818	848,482	877,849	243,195	363,623	894,313
**Average**	32,314.4	41,250.6	49,527	48,420.8	79,425.6	99,745	639,487.4	879,254.6	907,120.4	231,448	364,369.8	895,592.4

## Abbreviations

The following abbreviations are used in this manuscript:

MOCHs	Markov bi-directional chain model-based optimal cluster heads
$C_{MNs}$	Cluster member nodes
NACM	Non-associated cluster members
ACM	Association of cluster member
$R_{CHs}$	Random cluster heads
$W_{CHs}$	Waiting cluster heads
$O_{CHs}$	Optimal cluster heads
WSN	Wireless sensor network
SP	Scheduling phase
DT	Data transmission
